# Neural Cell Type Diversity in Cnidaria

**DOI:** 10.3389/fnins.2022.909400

**Published:** 2022-05-24

**Authors:** Simon G. Sprecher

**Affiliations:** Department of Biology, University of Fribourg, Fribourg, Switzerland

**Keywords:** nervous system, evolution, neurotransmitter, Cnidaria, *Nematostella*, *Hydra*, *Clytia*

## Abstract

Neurons are the fundamental building blocks of nervous systems. It appears intuitive that the human brain is made up of hundreds, if not thousands different types of neurons. Conversely, the seemingly diffuse nerve net of Cnidaria is often assumed to be simple. However, evidence that the Cnidaria nervous system is indeed simple is sparse. Recent technical advances make it possible to assess the diversity and function of neurons with unprecedented resolution. Transgenic animals expressing genetically encoded Calcium sensors allow direct physiological assessments of neural responses within the nerve net and provide insight into the spatial organization of the nervous system. Moreover, response and activity patterns allow the characterization of cell types on a functional level. Molecular and genetic identities on the other hand can be assessed combining single-cell transcriptomic analysis with correlations of gene expression in defined neurons. Here I review recent advances on these two experimental strategies focusing on *Hydra*, *Nematostella*, and *Clytia*.

## Introduction

**FIGURE 1:**
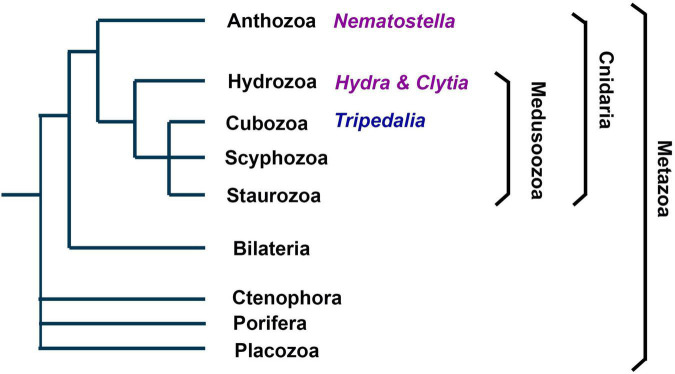
Phylogenetic position and main clades of Cnidaria. As metazoans Cnidaria are the sister group of bilaterians. They comprise the subphylums Anthozoa and Medusozoa, each consisting of several clades. Anthozoa are thought to be the earliest branch among Cnidaria and do not have a medusoid life stage. *Nematostella vectensis* belongs to Anthozoa. *Hydra* and *Clytia* belong to Hydrozoan, which belong to the Medusozoa.

The nervous system provides various critical functions throughout the life of animals. Sensory neurons allow us to perceive information about the environment, while motoneurons innervate muscles and control movements. Depending on the complexity of a nervous system there may be numerous interneurons linked in-between input and output neurons, which in turn are at the core of various types of computations that occur in neural circuits, such as the integration of information or the formation of memories. However, not all animals have neurons. Two early branching metazoan phyla, Sponges and Placozoans, do not have *bona fide* neurons, even though the molecular machinery, which is required for a functioning nervous system is largely present. Ctenophores on the other hand do have a nervous system (Whelan et al., 2017; Sachkova et al., 2021). The phylogenetic position of these early branching metazoans remains heavily debated and thereby also to some degree the origin of neurons. However, there is wide consensus that Cnidaria are the sister group of Bilateria (Moroz et al., 2014; Moroz, 2015; Whelan et al., 2015; Burkhardt and Sprecher, 2017; Feuda et al., 2017; Simion et al., 2017; Turner, 2021). Importantly, Cnidaria do have a nervous system. While the cnidarian nervous system is often termed a “simple nerve net” there is currently little evidence that the cnidarian nervous system is indeed simple. Cnidaria are typically radially symmetrical and therefore only contain an oral-aboral axis, defined by the single opening to the gastric cavity, often referred to as mouth opening. However, several species—including *Nematostella*, display an additional axis, termed directive axis and are therefore bilateral symmetrical (Saina et al., 2009). Cnidaria do not show a higher degree of nervous system centralization and do not possess a ganglia in a classical sense (Sarnat and Netsky, 2002; Hombria et al., 2021). However, the lack of ganglia should not directly imply that the body is covered with a uniform, diffuse nerve net. Depending on the species there may be different degree of complexity and condensation of neurons. Many species contain typically a prominent nerve ring surrounding the mouth opening, they may also have radial nerves and even ganglion-like aggregation of neurons. One of the best studied, complex neural organs of Cnidaria are the rhopalia, a sensory structure found in cubozoans. The rhopalium contains two lens eyes, as well as two pairs of simpler pit eyes and a statocyst (Nilsson et al., 2005). Anatomical studies in *Tripedalia cystophora* showed that the rhopalial nervous system contains over 1,000 neurons, which can be further divided into several morphologically different cell types, some defined by the expression of neuropeptides (Nielsen et al., 2021). While architecture of the neural circuits remains still largely unknown it has been shown that *Tripedalia cystophora* use visual cues from the surrounding world to alter its swimming behavior and thereby navigates in mangrove areas by using terrestrial cues seen through the water surface (Petie et al., 2011; Garm et al., 2012). The example of the rhopalial nervous system of cubozoans shows that in certain cnidaria species the nervous system may have evolved structures that are much more complex than the widely assumed simple nerve net (Petie et al., 2011; Garm et al., 2012; Nielsen et al., 2021). While one may argue that medusae have an overall more complex lifestyle since they are freely swimming, also the behavioral capacity of polyps may be more complicated than naively expected. The freshwater polyp *Hydra* has been studied particularly for its regenerative capacity for over a century (Galliot, 2012). *Hydra* has been shown to display an array of different behaviors, including rather complex behaviors, such as somersaulting, during which the animal detaches its foot and “stands” on its head, while moving the foot to become attached somewhere else (Passano and McCullough, 1962; Lenhoff, 1968; Han et al., 2018). Such types of behaviors require coordination of certain behavioral motives, which are most likely associated with the transition of the use of different neural networks. However, whether there are commonalities in circuit organization between different clades or between medusae and polyp remains largely unexplored. Similarly, the molecular nature and functional impact of different neuron types is not well understood. A first line of exploration in cell-type diversity in the nervous system comes from single-cell transcriptomics studies. The approach allows to analyze the molecular fingerprint of different cell types and to virtually investigate gene expression profiles. By linking single-cell sequencing data with reporter gene expression or *in situ* hybridization techniques it is possible to connect cell types and their identities. While such an approach requires the availability of genomic or transcriptomic data, the decrease in costs to establish a reference genome or transcriptome opens the approach for various species. The phylum Cnidaria contains several clades, that differ substantially in their life cycle and body plan (Figure 1). Much of the knowledge on the cnidarian nervous system stems from a few experimental species for which molecular and genetic techniques are established or emerging (Kelava et al., 2015; Bosch et al., 2017; Rentzsch et al., 2017; Helm, 2018; Rentzsch et al., 2019; Frank et al., 2020; Vogg et al., 2021; Houliston et al., 2022). Apart from *Hydra*, which was already mentioned these lab bread species include the starlet sea anemone *Nematostella vectensis*, the moon jellyfish *Aurelia aurita*, the jellyfish *Clytia hemisphaerica*, species of the hydrozoan genus *Hydractinia* (Figure 1; Houliston et al., 2010; Albert, 2011; Rosengarten and Nicotra, 2011; Technau and Steele, 2011; Matveev et al., 2012; Plickert et al., 2012; Layden et al., 2016; Rentzsch and Technau, 2016; Bosch et al., 2017; Frank et al., 2020; Rottinger, 2021; Nicotra, 2022). Substantial work has identified core developmental pathways in early neurogenesis, which appear to make use of a similar set of transcription factors as in bilaterians including members of the atonal and SoxB families (Layden et al., 2012; Kanska and Frank, 2013; Richards and Rentzsch, 2015; Flici et al., 2017; Gahan et al., 2017; Rentzsch et al., 2017). Much less is known regarding the processes involved in terminal differentiation, circuit assembly as well as the genetic diversity of neurons and their precise functions. In the current review I will focus on recent insights into the cell type diversity on a genetic level and links to functional roles.

## Organization of the Cnidarian Nervous System

At larval stages the nervous system has been used to study development studies for instance in *Nematostella, Clytia* as well as in *Hydractinia* (Leclere et al., 2012; Flici et al., 2017; Gahan et al., 2017; Rentzsch et al., 2017). However, the organization of the life cycle differs between distinct cnidarian clades. Mature animals may either have the form of either medusa or polyp (Technau and Steele, 2011). While the homology between the two body plans remains debated the gross organization of the nervous systems displays similarities (Rentzsch et al., 2019). In both cases there is typically a nerve ring surrounding the mouth opening and a more diffuse nerve net covering most parts of the body (Rentzsch et al., 2019). An intriguing feature of cnidarian synapses is that they are not necessarily unidirectional meaning that in in these cases neuronal signals may spread in both directions. Apart from the superficially diffuse nerve net there may be organized nerves running along the mesenteries in *Nematostella*, an endodermal structure containing muscles as well as gonads (Tucker et al., 2011). However, it remains largely unknown how diverse the neurons in the nerve net in fact are. Some insight into the organization and potentially diversity stems from studies using behavioral experiments in combination with genetically encoded Calcium sensors in *Hydra* and *Clytia*.

*Hydra* displays a series of different behaviors including contracting or bending and tentacle swaying. A recent machine learning based approach allowed a deeper characterization of six different basic behaviors: silent, elongation, tentacle swaying, body swaying, bending, contracting, and feeding (Han et al., 2018). Some more ground pattern behaviors appear more uniform such as bending, contracting, or extending. Somersaulting and feeding are more complex behaviors and consist of a series of individual behavioral motives. Such more complex behaviors require the animal to undergo a specific series of behavioral motives in a specific order, which in turn requires transition of different pre-motor and motor-programs in the nervous system. While they may be seen as not particularly challenging on a computational level, it nevertheless means that the neural circuits or circuits elements have to be able to execute the motor-programs in consecutive fashion and have to be stereotypic in a way to achieve the required outcome, but flexible to adapt for variation of the outcome. Interestingly in the case of *Hydra* semi-independent neural circuits have been identified based on activity patterns using the genetically encoded Gcamp sensor (Dupre and Yuste, 2017). Activity recording showed that different ensembles of neurons are active during different types of behaviors. For instance, an ensemble of neurons is active during elongations, termed Rp1 (Rp for rhythmic potentials), while another ensemble is active during radial contractions, termed Rp2. Such findings show that the nerve net in polyps is made up of different circuits which are required during different behaviors.

In medusae the umbrella contains both a nerve net as well as different types of muscles, thereby controlled deformations of the umbrella can be directly used for different types of behaviors including swimming and feeding. *Clytia* medusa show a folding behavior of the umbrella, which thereby facilitates the transfer of caught plankton from the tentacle to the mouth. Neurons expressing the Fmrfamide peptide are involved in this behavior (Weissbourd et al., 2021). Using transgenic animals expressing the nitroreductase gene, bi-cistronically with Rfp, allowed to specifically kill Fmrfamide neurons when providing the drug metronidazole, while monitoring the removal of cells using Rfp. In animals lacking Fmrfamide neurons the folding behavior is completely lost. Using another transgenic line that expresses the genetically encoded Calcium sensor Gcamp in Fmrfamide expressing neurons shows that these cells are indeed active during the folding behavior. Interestingly, by monitoring the Gcamp activity patterns in more detail it became evident that there are different, discrete ensembles of neurons that appear to make up the entire Fmrfamide population in the umbrella (Weissbourd et al., 2021). Thus, also in medusae different neural subcircuits exist, that are critical for specific aspects of behavior.

These examples show that the nerve net is not uniform, and that certain neuron types and certain ensembles of neurons are required for distinct motoprograms driving behavior. It furthermore demonstrates that there is a spatial organization and subdivision of a certain, molecularly defined neuron type. On a technical level these approaches highlight the impact of two comparably simple and widely used neurogenetic tools in many model organisms: genetic cell-ablation and optic activity monitoring.

## Molecular Identities of Cnidarian Neurons

Functional features of neurons, such as propagation of membrane currents, transport of and packaging of neurotransmitter or the release of neurotransmitters at the synapse depend on a molecular level on series specific proteins. The expression of these genes encoding for these proteins may in turn be used as defining molecular markers. Such markers typically include synaptic proteins, enzymes in neurotransmitter synthesis, neuronal cell adhesion proteins, but also other pan-neuronal markers, for which the function is less well understood. The Rna binding protein Elav belongs to the latter group. Initially identified in the fruit fly, Elav family proteins have been shown to be expressed in neurons of vertebrates, but also in Cnidaria (Marlow et al., 2009; Nakanishi et al., 2012). The conserved expression in neurons further suggests a common function of these proteins in the nervous system. In *Nematostella vectensis* a transgenic Elav-mOrange reporter is widely expressed in the nervous system, but the line appears to not express pan-neuronally since other neuronal reporter lines are not completely overlapping with the Elav-mOrange reporter (Nakanishi et al., 2012). Single-cell transcriptomics also identified Elav as neuronal marker in *Clytia* (Chari et al., 2021). The pan-neuronal *Hydra* Gcamp line described above made use of an *actin*-promotor, a gene family that is not exclusively expressed in the nervous system, but cytoskeletal genes are often strongly enriched in the nervous system and some genes may contain nervous system specific enhancers (Dupre and Yuste, 2017). Widely used antibodies for the nervous system include for instance anti acetylated-tubulin or anti tyrosinated tubulin antibodies. Other typical markers that are either specific or highly enriched in many neuron types includes certain proteins for synaptic transmission, such as Synaptobrevin, Synaptotagmin, Synapsin, Homer, or Synaptophysin.

Such common neural markers genes are important features in single-cell transcriptomic studies and help identify neural cell clusters. Recent single-cell transcriptomic analyses were performed on *Nematostella*, *Hydra*, and *Clytia*, investigations more species are very likely underway. The characterization of clusters identified by single-cell transcriptomic approaches and how they may be linked to cell-fates or cellular identities resulted in a broad, rather vivid discussion in the community on what cell types indeed are, how—or even if- clear boundaries cell-types can be drawn, how developmental transitions should be assessed and how in a broader sense the cell-state should be defined. It is important in this context to note that cell clusters in single cell analyses may comprise more than one cell type and that the boundaries ultimately largely depend on sampling depth and to some degree also on conscious decisions when analyzing the data. Nevertheless, the findings of single-cell studies are powerful and extremely informative. In *Nematostella* 32 cell clusters were identified by restricted expression or enrichment of specific genes comprising both larval and adult tissues (Sebe-Pedros et al., 2018). These genes include several transcription factors such as *Pou4*, *Rx*, *FoxD*, *FoxL2*, *SoxC*, or Six1/2. Identification of marker genes in single-cell transcriptomic analyses can indeed be powerful approach for addressing neural identity a transgenic reporter for *FoxL2* shows expression in neurons and cnidocytes and a reporter for *otxC* in neurons. While molecular or functional features of these neural types and a putative role for the behavior of the animals remains currently unexplored these examples show that combining single-cell analysis with subsequent enhancer analysis of marker genes provides a powerful technical approach for neurogenetic studies. Other neural cluster markers in *Nematostella* include G-protein coupled receptors, ion channels and candidate neuropeptide precursors genes. In a *Hydra* single-cell transcriptomic experiment 15 nervous system associated clusters were identified, three containing neural progenitors and 12 consisting of differentiated neuron subtypes (Siebert et al., 2019). Markers specific or enriched in defined clusters include *Lwamide*, *Cnot*, *Innexin2*, *Ndf1*, and *Alpha-Ltx-Lhe1a-like*. To determine differential expression of genes in endodermal and or ectodermal nerve net a TagSeq based experiment allowed to differentiate clusters that belong to the two domains. Gfp reporter transgenes for Ndf1 and Alpha-Ltx-Lhe1a-like indeed allowed a differentiation and showed that *Ndf1*-Gfp is expressed in endodermal neurons, while *Alpha-Ltx-Lhe1a-like*-Gfp is expressed in ectodermal neurons. This example elegantly shows that a further assignment of clusters within defined spatial domains of the nervous system can be achieved. Single cell transcriptomic analysis of *Clytia* identified 14 neural clusters as well as a cluster of neural progenitors defined by the expression of the bhlh transcription factor *Neurogenin (Chari et al., 2021)*. As in *Nematostella* and *Hydra* the expression of specific or enriched markers include transcription factors such as *Six-like*, *Sox10*, and *Hlh6* as well as G-protein coupled receptors *agpcr3* and *agpcr2*. Interestingly, the neuropeptide precursor genes *Pp11* and *Pp5* also belong to these markers. *In situ* hybridization of six neuropeptide precursor (Pp11, Pp5, Pp25, Pp17, Pp20, and Pp7) show marked expression in different subpopulations of neurons, which correlates with data from the scrnaseq study. Interestingly also in *Hydra* a series of highly specific regionalized neuropetides, and thereby likely neural subtypes, were described using *in situ* hybridization (Noro et al., 2019).

While single-cell analyses directly provide insight into the molecular fingerprint of neurons and thereby open direct avenues for future genetic studies, it is important to note that these approaches can also immediately be used to go beyond mapping genes to cells *in silico*. An intriguing example are findings on developmental trajectories of different cell types. Whole animal or whole organ single-cell transcriptomics often contain progenitor cells, differentiated cells and mature cells. Developmental transitions are typically transient and different degrees between immature and mature cells can be observed. Several approaches have been developed to investigate these including Monocle, Rna velocity and Slingshot (La Manno, et al., 2018; Street et al., 2018; Cao et al., 2019). In *Hydra* well studied multipotent interstitial stem cells (Iscs) have been shown to be at the center of developmental and regenerative processes. Interestingly, single-cell transcriptomic analysis shows different intermediate progenitor cell types and allowed to reconstruct two putative trajectories originating from a *Hy-icell1* expressing stem cell population into first two types of *HvSoxC* expressing cells (Siebert et al., 2019). These different lineages appear to be ectoderm and endodermal pathways. It is interesting to note that in either lineage go through a Myb-expressing progenitor, suggesting that the neurogenic program is comparable. Another interesting finding is that these analyses corroborate that nematocytes, gland cells, and neurons are closely related in their linage origin. This core developmental relationship was found in *Clytia*, *Nematostella*, and *Hydra*. There are a few interesting points that can be drawn from this notion. First, some features of nematocytes display similarities with sensory cells. They are a highly derived and cnidaria-specific cell type they are activated only touch. Moreover, nematocytes firing is not a purely mechanical reflex as it not elicited if for instance two tentacles touch each other. Interestingly gene expression analysis of isolated nematocytes of *Nematostella* showed that *Cnido-Jun* and *Cnido-Fos1* are enriched in these cells, two genes which are widely used in neurosciences as immediate early genes depicting neuronal activity (Sunagar et al., 2018). The actual stinging process is an ultrafast exocytosis of the extrusive organelle, which is Calcium-dependent, similarly to synaptic exocytosis. However, if and how nematocytes use neuron-like molecular mechanisms in their cellular function and how they are connected to the nervous system remains still largely unexplored. Second, also gland cells display molecular similarities with neurons, including the fact that they can use controlled exocytosis to release certain vesicles.

While the use of neuropeptides is an important and global feature of neural communication, the main form of chemical synaptic communication is thought to depend on small molecule neurotransmitters. These neurotransmitters include Glutamate, Acetylcholine, and Gaba. Markers for a specific neurotransmitter identity are either the detection of the neurotransmitter molecule itself as well as the genes or proteins for are enzymes that are involved in the biosynthesis of the neurotransmitter or vesicular neurotransmitter transporters. While most enzymes required for the synthesis of neurotransmitters are present in Cnidaria their function is not well studied. Single-cell analysis in *Clytia* shows that the closest homologs of the vesicular Glutamate transporters are either expressed in nematocysts or non-neuronal cells. Interestingly *vGlut* genes of *Clytia* lack a conserved and critical arginine residue suggesting that vesicular release of Glutamate may not be occurring. Moreover, glutamate decarboxylase (Gad), which is critical in Gaba biosynthesis appears to be restricted to gastrodermal cells (Chari et al., 2021). Interestingly *in situ* hybridization studies in *Nematostella* showed that the core enzymes involved in the biosynthesis of Glutamate, Acetylcholine and Gaba also appear to be predominantly expressed in non-neuronal tissues (Oren et al., 2014). Application of the Gaba receptor agonist baclofen during development in *Nematostella* appears to have inhibitory effects on neurogenesis further suggesting a non-neuronal, but rather a developmental role of Gaba receptor signaling (Levy et al., 2021). In *Nematostella* anti-Gaba immunostaining showed expression in neurons associated with the pharynx and putatively sensory neurons. Taken together these findings raise the question of what in fact common and widely used neurotransmitters in Cnidaria are.

## Structure of Cnidarian Neurons

**FIGURE 2:**
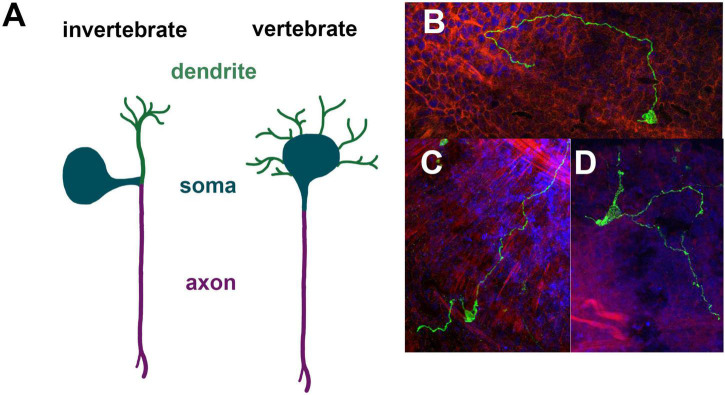
Morphological properties of cnidarian neurons. Prototype invertebrate neuron with a unique primary neurite compared to the prototype vertebrate neuron with a single axon and multiple dendritic arbors **(A)**. Unipolar, bipolar and tripolar neurons in *Nematostella* expressing Elav-mOrange in mosaic animals **(B–D)**.

The cellular morphology of neurons is diverse. Thus, while features are shared between neurons, there is no clear neural prototype. A core feature of neurons is the existence of neurites, which in many cases can be divided into dendrites and axons. Dendrites typically are shorter and may loosely be regarded as input regions, while axons are long and provide output. However, this input-output relationship of dendrites and axons is clearly too simplistic to consider the diverse pre- and post-synaptic interactions between neurons. Depending on the number of neurites which originate from the soma different cellular morphologies can be defined such as monopolar neurons (one neurite), bipolar neurons (two neurites), tripolar neurons (three neurites), and quadripolar neurons (four neurites). Typically, invertebrate and vertebrate neurons differ substantially in their gross cellular morphology (Smarandache-Wellmann, 2016). The prototype vertebrate neuron has one long, unbranched axon and several short, branching dendritic arbors. Physiologically the synaptic integration occurs in dendrites and soma and the action potential is thought to be initiated from the axon hillock and propagating away from the soma. In invertebrate ganglia the cell bodies are often removed from the neuropil in a fashion that a single neurite extends from the soma, which then branches into axons and dendrites (Figure 2). A prototypic neuron would have a bifurcation of the primary neurite into a single, unbranched axon and a dendritic arbor, which can further branch out (Smarandache-Wellmann, 2016). This rather simplistic depiction provides some morphological framework to assess cnidarian neurons. As mentioned above a quite unusual feature of cnidarian synapses is that they may be bidirectional in their organization. This of course complicates conceptually how flow of information in a network is coordinated. Currently there is little information about the precise make up and wiring of neurons in cnidarian nerve nets. Moreover, how general this notion is in *Hydra*, *Nematostella* and *Clytia* or which types of neurons show non-polar synapses remains unknown.

In the past morphological features have been used to assign a certain type to some neurons (Figure 2). In *Nematostella*, typically the location of a neuron was noted and if it is associated with a specific organ as well as the number of neurite extensions. For instance, neurons expressing the neuropeptide Lwamide have been characterized using a Lwamide-mCherry reporter line (Havrilak et al., 2017). There are five morphologically distinct neuron types: longitudinal neurons, tripolar neurons, mesentery neurons, pharyngeal neurons and tentacular neurons. Different branching patterns have also been defined as type 1 ganglion cells for bipolar neurons, type 2 ganglion cell for tripolar neurons and type 3 ganglion cells for quadripolar neurons (Marlow et al., 2009). However, it is worth noting that the branching pattern is currently defined by the branching from the soma and possible branching of longer axons have not been further assigned.

## Perspective

Cnidaria are a diverse group of animals, which have adapted to various different aquatic environments including the deep sea, polar regions, tropical, and temperate seas as well as freshwater ecosystems. They can be colonial or solitary and propagate sexually or asexually. From a neurobiological perspective we know surprisingly little about this animal clade, even though they are placed at a particularly relevant phylogenetic position as sister group to bilaterians. Much of our general knowledge about nervous systems stems from vertebrates and a few invertebrate clades. The comparison between canonical models, such as mouse, zebrafish, *Drosophila*, and *C. elegans* highlight commonalities and differences between nervous systems. However, despite the use of different bilaterian models, much about the fundamental features and origin of nervous systems remains unknown. Cnidaria are here in the unique position to provide answers. The advent of high-throughput sequencing and emergence of genomes of different Cnidaria species has already provided critical information of the genomic make-up of the nervous system on a molecular level. Adding single-cell transcriptomic data has provided a much-needed additional degree of resolution, thus allowing us to peek into the diversity of neuron types and providing ample opportunity for hypothesis driven research. In particular the possibility for transgenesis and genome editing in several species further opens up avenues for detailed genetic and mechanistic studies. Many of our general assumption on neurons function get corroborated, but there are also many areas that bring surprises. Until today most molecular and genetic studies focus on neurogenesis and nervous system development. The tools to assess gene function of behavior and to move toward neurogenetics are there in several species. While I here focus on *Hydra*, *Nematostella* and *Cyltia* it is worth mentioning that transgenesis and Crispr genome editing has also been achieved in *Hydractinia* (Kunzel et al., 2010; Gahan et al., 2017; Sanders et al., 2018; Chrysostomou et al., 2022). It is fair to assume that in the coming years studies, such as the ones discussed here on *Hydra*, *Nematostella*, *Cyltia, Hydractinia, Aurelia*, and possibly other species will provide important insights into the function of nervous systems in Cnidaria, but also much broader into nervous system evolution.

## Author Contributions

The author confirms being the sole contributor of this work and has approved it for publication.

## Conflict of Interest

The author declares that the research was conducted in the absence of any commercial or financial relationships that could be construed as a potential conflict of interest.

## Publisher’s Note

All claims expressed in this article are solely those of the authors and do not necessarily represent those of their affiliated organizations, or those of the publisher, the editors and the reviewers. Any product that may be evaluated in this article, or claim that may be made by its manufacturer, is not guaranteed or endorsed by the publisher.
